# Application of Atmospheric and Room-Temperature Plasma (ARTP) to Microbial Breeding

**DOI:** 10.3390/cimb45080408

**Published:** 2023-08-04

**Authors:** Qin Zhang, Renyun Miao, Rencai Feng, Junjie Yan, Tao Wang, Ying Gan, Jin Zhao, Junbin Lin, Bingcheng Gan

**Affiliations:** 1 Institute of Urban Agriculture, Chinese Academy of Agricultural Sciences, Chengdu 610299, China; zhangqin@caas.cn (Q.Z.); miaorenyun@caas.cn (R.M.); fengrencai@caas.cn (R.F.); yanjunjie@caas.cn (J.Y.); wangtao03@caas.cn (T.W.); ganying@caas.cn (Y.G.); zhaojin@caas.cn (J.Z.); linjunbin@caas.cn (J.L.); 2 Chengdu National Agricultural Science and Technology Center, Chengdu 610299, China

**Keywords:** ARTP, application, bacteria, fungi, microalgae

## Abstract

Atmospheric and room-temperature plasma (ARTP) is an efficient microbial mutagenesis method with broad application prospects. Compared to traditional methods, ARTP technology can more effectively induce DNA damage and generate stable mutant strains. It is characterized by its simplicity, cost-effectiveness, and avoidance of hazardous chemicals, presenting a vast potential for application. The ARTP technology is widely used in bacterial, fungal, and microalgal mutagenesis for increasing productivity and improving characteristics. In conclusion, ARTP technology holds significant promise in the field of microbial breeding. Through ARTP technology, we can create mutant strains with specific genetic traits and improved performance, thereby increasing yield, improving quality, and meeting market demands. The field of microbial breeding will witness further innovation and progress with continuous refinement and optimization of ARTP technology.

## 1. Introduction

In the context of rapid societal progress, microorganisms are experiencing a remarkable surge in their applications across diverse industries, encompassing food, pharmaceuticals, energy, and agriculture. Notably, their pivotal role in bioremediation, which involves the utilization of microorganisms to restore polluted environments, cannot be overstated. In modern society, microorganisms have emerged as indispensable assets, effectively meeting the escalating demand for microbial products.

To fully harness the potential of microorganisms and address the increasing demand, the cultivation of superior strains has become imperative in the realm of microbial applications [1]. Various methods are employed to breed microbes, including natural screening, gene recombination, mutation breeding, metabolic control breeding, and genetic engineering breeding [2,3,4]. Among these methods, mutation breeding induces various biological effects, such as base deletion, chromosome breakage, and gene recombination, leading to altered traits in the offspring and the introduction of numerous mutations [5,6].

In particular, directional screening serves as a valuable supplementary tool for mutagenesis breeding, enabling the swift identification of desired target traits and facilitating the acquisition of microbial strains possessing the desired characteristics. Such traits can greatly benefit the microbial strain, resulting in improved qualities like higher yields, increased resistance to environmental stress, and enhanced nutritional values. It is worth noting that genetic modification techniques have emerged as powerful tools for agriculture and biotechnology applications.

Mutation breeding commonly employs two main approaches: physical mutagenesis and chemical mutagenesis [7]. Physical mutagenesis involves subjecting organisms to high-energy radiation, such as gamma rays, X-rays, UV light, neutrons, ion beams, beta particles, alpha particles, and plasma [8]. These forms of radiation induce genetic mutations by causing DNA damage. On the other hand, chemical mutagenesis involves the use of various chemicals to induce genetic changes. Chemical mutagens can damage DNA through processes like alkylation or the formation of base compounds [9]. Examples of chemical mutagens include alkylating agents, natural base analogs, lithium chloride, nitroso compounds, azides, base analogs, antibiotics, hydroxyamine, and acridine.

However, both radiation (e.g., X-ray and γ-ray) and chemical mutagenesis methods exhibit certain limitations. Radiation mutagenesis suffers from challenges in accurately controlling dosage and yields a low mutation rate. Furthermore, the generation of radiation necessitates bulky and costly equipment, specialized operational expertise, and additional safety precautions to restrict its usage. On the other hand, chemical mutagenesis, despite offering a higher mutation frequency, generates toxic agents that are arduous to eliminate, thereby posing substantial risks to both users and the environment.

Against this backdrop, atmospheric and room-temperature plasma (ARTP) has emerged as an innovative and efficient physical mutagenesis method. The ARTP system was originally developed by the Department of Chemical Engineering at Tsinghua University and subsequently commercialized by Si Qing Yuan Biotechnology (now Tmax Tree Co., Ltd., Jiangsu, China). Following its commercialization, the research team at Tsinghua University conducted comprehensive investigations into the mutagenesis mechanism, confirming the exceptional mutagenic efficiency of this technology through extensive fundamental studies [10,11]. ARTP surpasses traditional chemical and radiation mutagenesis by inducing high DNA damage and offering a greater diversity of mutations [12]. Moreover, it proves effective at creating mutations in genes that are difficult to target using other methods. Additionally, ARTP enables the creation of mutations in multiple genes simultaneously, facilitating faster and more efficient gene editing. Notably, the primary gas used in ARTP systems is helium, and it does not generate hazardous materials [13].

### 1.1. Principle of ARTP

ARTP technique has garnered considerable attention for its capacity to induce mutations in microorganisms. Operating at atmospheric pressure and temperatures ranging from 25 to 40 °C, ARTP generates plasma jets consisting of helium atoms, oxygen atoms, nitrogen atoms, and OH radicals. Plasma is commonly referred to as the “fourth state” of matter, in addition to solid, liquid, and gas. Extensive studies have revealed that the active particles present in plasma can cause structural changes to microbial cells and membrane walls, resulting in damage to microbial genes and significant alterations in their sequence and metabolism [14].

**Figure 1:**
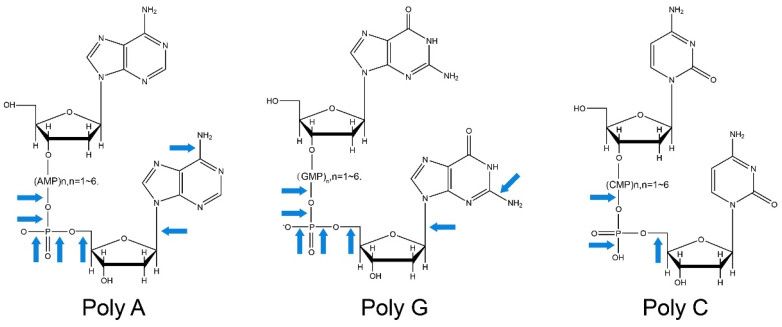
The structure of dN_8_ was damaged by ARTP [10]. The arrows indicate the bonds that would have been cleaved to produce fragments with the assigned molecular weights in the mass spectrum.

ARTP offers several advantages over traditional mutagenesis methods. Firstly, it inflicts substantial damage to DNA, resulting in a high mutation rate in microorganisms. The resulting mutations can be inherited by subsequent generations, giving rise to stable mutant strains (Figure 1) [10]. Moreover, ARTP outperforms molecular biology technologies, in terms of simplicity, cost-effectiveness, and the absence of hazardous or damaging chemicals.

The ARTP technology serves as a mutagenesis method based on discharged plasma. Within the ARTP system, high-purity helium gas (≥99.99%) is ionized by an externally applied RF electric field, leading to the formation of a non-thermal plasma jet downstream of the plasma nozzle exit [5]. These high-energy and highly reactive particles, including excited helium atoms, oxygen atoms, nitrogen atoms, and OH radicals, transiently irradiate microorganisms, leading to genetic variation and mutation. The active energy particles in ARTP cause damage to the genetic material of microbial strains and induce the initiation of the SOS repair mechanism in biological cells [15,16]. SOS repair is a mechanism prone to errors, involving mismatch repair and recombination repair, which give rise to diverse mismatched sites and ultimately stabilizes genetic alterations, leading to the emergence of mutant strains. SOS repair preserves genome integrity and enhances cell survival. The intensity of SOS repair is intricately linked to the magnitude of DNA damage. The UMU-test method shows that ARTP has significant effects on the genetic material of organisms, with a rich range of damage mechanisms, especially on the genetic material of eukaryotes such as chromosomes [12]. Therefore, ARTP exhibits improved mutagenic performance and a broader range of applications than other mutagenesis methods. Genomic sequencing reveals that mutant strains obtained through ARTP mutagenesis have a greater number of gene mutation sites [17]. This highlights the potential of ARTP as a valuable tool for genetic research and breeding purposes.

### 1.2. Technical Parameters

The applicability of ARTP mutagenesis to various microorganisms allows for a wide range of parameter combinations. The key parameters and their importance are summarized as follows: First, strain vitality: opt for strains in the logarithmic growth phase as the mutagenesis material, as cells proliferate rapidly and are more sensitive to external environmental factors during this growth stage [18]. Second, cell concentration: select an appropriate concentration of cell suspension for mutagenesis. Third, mutagenesis dose: The ARTP mutagenesis dose is closely related to the mutagenesis time. Typically, the mutagenesis dose that leads to a 90% cell death rate is used as a parameter, as it results in a relatively high forward mutation rate [19]. Fourth, subsequent dilution: to fully utilize the agar plate surface and obtain single, dispersed colonies, properly dilute the treated cell suspension and then spread it evenly on the agar plate surface. Lastly, screening method: Different colony screening methods can be chosen based on the specific research objectives. For instance, Yu et al. [20] developed a high-throughput screening method, utilizing streptomycin resistance prescreening and a 24-well deep well plate/microplate reader rescreening strategy, to identify a high-yield neomycin sulfate mutant strain from a large number of mutants. Subsequently, through fermentation condition optimization, the neomycin sulfate concentration of the strain Sf6-2 was increased by 100%. Similarly, Yao et al. [21] identified 10 high-yield tylosin-producing strains from a mutant library of *S. fradiae* using a high-throughput 24-well plate screening method and UV spectrophotometry. Further, re-screening in shake flasks led to the identification of two strains with significantly higher tylosin A production than the wild-type strain. The optimization of these parameters is crucial for achieving successful ARTP mutagenesis, obtaining desired mutations, and effectively harnessing the potential of this technique for microbial breeding.

### 1.3. Mutagenesis Targets

**Figure 2:**
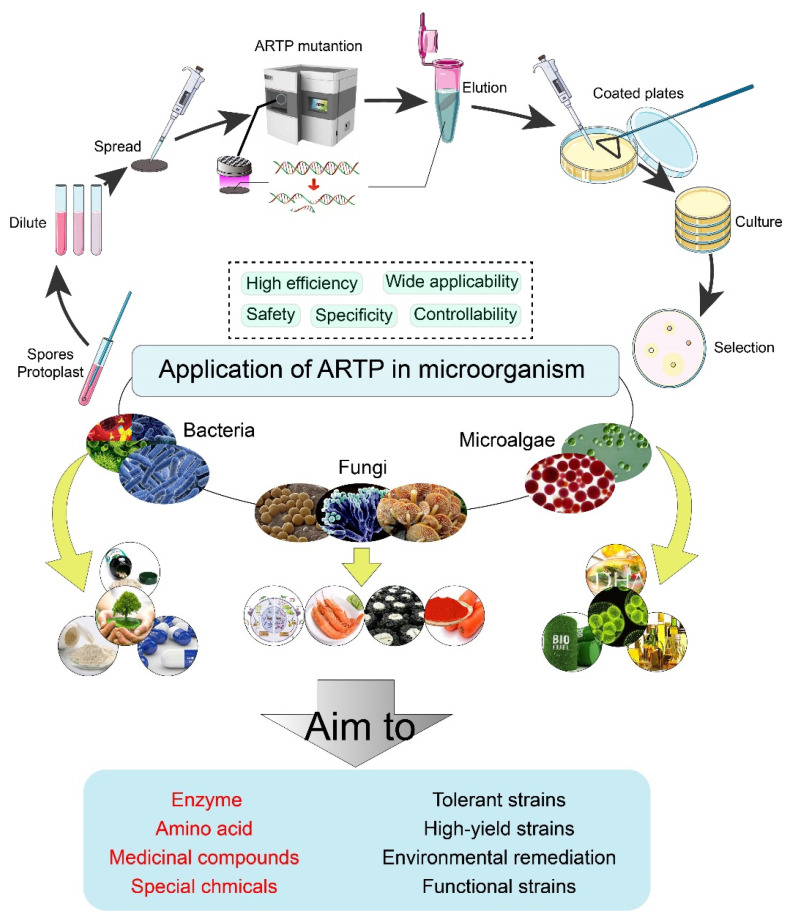
Application of ARTP in microorganisms.

The utilization of ARTP in mutagenesis holds great potential for producing superior strains of microorganisms with enhanced traits. This technique has demonstrated its effectiveness across various microorganism groups, including bacteria [22], fungi [23], and microalgae [24]. The success of ARTP can be contributed to several key factors, including its high efficiency in inducing mutations, its capacity to cause diverse genetic damage, and its operation under mild and safe conditions (Figure 2).

Through the application of ARTP, researchers can introduce specific changes to the genetic material of microorganisms, leading to the development of desirable traits. This method has proven particularly advantageous in improving critical microbial characteristics, such as increased product yield, enhanced stress tolerance, or optimized metabolic pathways. Consequently, mutagenic strains present a promising option for the industrial production of various desirable active substances. This article provides a concise overview of the applications and future prospects of ARTP technology in the breeding of microorganisms. 

## 2. Application of ARTP to Bacteria

ARTP technology has wide-ranging applications in bacteria, offering significant benefits in improving bacterial strains, as well as enhancing their yield and quality. Bacteria are involved in the production of various valuable products, including enzymes, amino acids, antibiotics, bacterial drugs, biofuels, and food additives (Table 1). ARTP serves as a powerful tool for engineering microbial cell factories, contributing to cost reduction and process optimization in production. As an essential tool for the biotechnology industry, the application of ARTP paves the way for a more sustainable and efficient means of producing diverse products.

### 2.1. Enzyme Overproducers

ARTP technology has emerged as a powerful tool for enhancing enzyme production and optimizing biocatalyst production. In the context of chitosan oligosaccharides production, ARTP has effectively been used to generate mutant strains of *Bacillus cereus*, resulting in increased chitosanase yield and activity [25]. Chitosanase, a crucial enzyme found in microorganisms, breaks down chitosan into smaller chitosan oligosaccharides, which possess valuable antimicrobial, antioxidant, and anti-inflammatory effects, making them promising for various applications [26].

Moreover, ARTP has played a pivotal role in obtaining high-yield thermostable protease-producing strains, such as *Bacillus licheniformis* TP1-5, which exhibit significantly enhanced enzyme activity compared to the original strain [27]. These thermostable proteases, known for their stability and activity at high temperatures, have practical applications in industries such as high-temperature washing, medicine, and leather production.

In addition to proteases, ARTP has been instrumental in improving Coenzyme Q10 (CoQ10) production in *Rhodobacter sphaeroides* [28,29,30,31,32]. Coenzyme Q10, an essential dietary supplement, plays a vital role in antioxidant processes and bioenergy generation. By employing ARTP mutagenesis and subsequent selection of mutant strains, the production efficiency of Coenzyme Q10 was significantly improved, achieving an impressive efficiency of 80.38%.

Furthermore, ARTP has been utilized to generate mutant strains of alkaline phosphatase-producing bacteria, such as *Bacillus amyloliquefaciens* S-52 [33]. The alkaline phosphatase activity of the mutant strain S-52 was determined to be 12,110.6 U/L, which is 4.67 times greater than that of the original strain. ARTP has also been employed in the production of other enzymes, such as protease [34,35], alginate lyase [36], α-amylase [37], and alkaline protease [38]. The mutant strains exhibited significantly higher enzyme production capacity compared to the original strain, demonstrating the effectiveness of ARTP in improving enzyme yield.

In conclusion, the applications of ARTP technology in enzyme production have shown promising results in various microbial strains. Its potential in enhancing enzyme yields, improving activity, and optimizing production processes has significant implications for diverse industries, contributing to advancements in biocatalysts and enzyme-based products. As ARTP continues to evolve and gain momentum, its integration into enzyme engineering and biotechnology promises to unlock new avenues for sustainable and efficient enzyme production.

### 2.2. Amino Acid Overproducers

ARTP technology has demonstrated remarkable efficacy in enhancing amino acid synthesis in various bacterial strains. Extensive research has showcased its versatile application in improving the production of specific amino acids, including poly-γ-glutamic acid (PGA), L-serine, L-glutamine, L-histidine, and L-isoleucine.

PGA, a biopolymer composed of glutamic acid monomers, exhibits diverse biological activities, and ARTP-induced mutations have successfully led to significant increases in poly-glutamic acid synthesis, enabling cost-effective production [39]. Similarly, L-serine, an essential amino acid with crucial roles in physiological processes and overall health, has benefited from ARTP technology, with researchers achieving notable improvements in L-serine production compared to parent strains [40].

The demand for L-glutamine, another essential amino acid, has prompted efforts to enhance its production using ARTP-mediated approaches. Liang et al. [41] combined mutagenesis with metabolic engineering to optimize L-glutamine production, presenting potential advantages across various applications. Additionally, Lv et al. [42] employed ARTP and gene editing techniques to develop a highly productive strain, CGQ03/pXMJ19-R5-*glnA^Sc^*-*ppk^Ec^*, derived from *Corynebacterium glutamicum* ATCC 14,067. Firstly, the strain ATCC 14,067 underwent ARTP mutagenesis, resulting in the isolation of a mutant strain designated as N01, which exhibited a significantly increased yield of L-glutamine. Subsequently, gene editing techniques, such as gene knockout and overexpression, were applied to manipulate essential genes involved in the L-glutamine metabolic pathway. These genetic modifications facilitated the enhancement of L-glutamine production in the strain CGQ03/pXMJ19-R5-*glnA^Sc^*-*ppk^Ec^*. The engineered strain exhibited remarkable L-glutamine yields, reaching up to 73.5 ± 3.1 g/L, representing a substantial improvement compared to the parent strain.

Moreover, ARTP technology has been successfully applied to induce mutations in *Corynebacterium glutamicum* strains, resulting in the development of mutant strains with heightened production of other amino acids such as L-histidine [43] or L-isoleucine [44].

The availability of high-quality amino acids holds immense value for the industrial and pharmaceutical sectors. By leveraging the power of metabolic engineering and ARTP technology, researchers can significantly enhance amino acid yields and optimize strain metabolic performance, effectively meeting the escalating demands of diverse industries. In conclusion, ARTP technology has emerged as a highly promising tool for enhancing amino acid synthesis in bacteria. Its successful application to improving the production of specific amino acids, including PGA, L-serine, and L-glutamine, presents an economically viable and sustainable approach to amino acid production, effectively catering to the needs of various sectors. The integration of metabolic engineering with ARTP technology further amplifies the potential for optimizing amino acid yield and strain performance, paving the way for novel advancements in this field.

### 2.3. Antibiotics Overproducers

ARTP technology has found extensive application in the production of antibiotics, particularly in the development of bacteriocins, which are biologically active peptides or proteins synthesized by ribosomes. Bacteriocins exhibit strong inhibitory effects against competing species and possess broad-spectrum antibacterial activity [45,46,47]. Through a combined mutagenesis approach, multiple high-yielding bacteriocin mutant strains were developed from *Lactobacillus plantarum*, with production enhancements ranging from 103.48% to 551% [48,49,50].

In the case of dalbavancin, a lipoglycopeptide antibacterial agent effective against various Gram-positive organisms, including those with multidrug resistance [51], researchers employed a composite mutagenesis approach combining UV and ARTP. The aim was to enhance the yield of the dalbavancin precursor in Nonomuria spp. Wang et al. [52] utilized the strain DW-3-19 as the initial strain and subjected it to UV mutagenesis under specific conditions, resulting in the isolation of the DW-4-127 mutant strain. The fermentation process of DW-4-127 exhibited a 33.5% enhancement in the production of the dalbavancin precursor compared to the starting strain. Subsequently, the mutant strain DW-4-127 underwent ARTP mutagenesis and was subjected to selection using 0.6 mg/L of streptomycin as the screening agent, leading to the acquisition of the high-yield strain DW-5-52. The dalbavancin precursor production of DW-5-52 demonstrated a significant 23.9% increase compared to DW-4-127 and an impressive 68.7% improvement compared to the starting strain. Furthermore, strain DW-5-52 exhibited favorable genetic stability.

Through mutagenesis and screening approaches, researchers can obtain mutant strains with enhanced antibiotic production and activity, thereby improving efficiency and yield in antibiotic production. As a result, ARTP technology holds great promise in the creation of more efficient and high-quality antibiotics, effectively addressing the growing demand in the medical and pharmaceutical sectors.

Continued improvement and optimization of ARTP technology will drive further innovation and progress in the field of antibiotic production. By leveraging ARTP’s capabilities, researchers can contribute to the development of novel antibiotics that are more effective, safer, and capable of avoiding antibiotic resistance, thus benefiting global healthcare and public health.

### 2.4. Environmental Remediation

ARTP technology is extensively used in environmental remediation, enabling the generation of mutant bacterial strains with enhanced pollutant degradation capabilities and tolerance, thus improving environmental remediation efficiency. For instance, Wang et al. [53] utilized *Bacillus amyloliquefaciens* A3 as a wild-type strain, which produces lipopeptide biosurfactant, to enhance biosurfactant production through ARTP mutagenesis, aiming to remove petroleum hydrocarbons from soil. In soil column leaching experiments, they achieved 45.44% removal of petroleum hydrocarbons by modifying relevant enzyme activities.

Similarly, Bao et al. [22] discovered *Bacillus velezensis* LYB-23, a chromium-resistant bacterium, from waste chromium residues. Employing the synergistic approach of domestication and ARTP technology, they successfully elevated the minimum inhibitory concentration of *B. velezensis* for chromium from 80 mg/L in the original strain to 400 mg/L in the mutant strain. This groundbreaking method significantly augmented the bacteria’s tolerance to Cr (VI) and improved its efficiency in reducing and absorbing this pollutant, showcasing its potential for breeding bacteria tailored for environmental remediation applications.

Furthermore, Yang et al. [54] isolated *Pseudomonas fluorescein*, a bacterium capable of producing extracellular polymeric substances (EPS), from soil. Harnessing ARTP technology, they obtained a mutant strain, named T4-2, with substantially enhanced EPS production and improved flocculating activity. The EPS generated by T4-2 showed a high adsorption capacity for chromium (VI) and has great potential for efficiently removing cadmium from contaminated soil or lake water, offering a promising solution for environmental remediation.

The utilization of ARTP technology in environmental remediation showcases its efficacy in enhancing pollutant degradation capabilities and tolerance of bacterial strains. This advancement provides opportunities to improve the efficiency and effectiveness of the environmental remediation processes, ultimately contributing to a cleaner and healthier environment.

### 2.5. Others

ARTP technology has diverse and significant applications in enzyme production, amino acids, antibiotics, and environmental remediation. Its role in agriculture and the breeding of resistant bacteria is also noteworthy. Plant growth-promoting (PGP) bacteria, which offer eco-friendly alternatives to chemical fertilizers, have been a focus of research in this regard.

For instance, Wang et al. [55] isolated *Pantoea* sp. YSD J2 from *Cyperus esculentus* L. var. sativus and employed ARTP mutagenesis to enhance indole-3-acetic acid (IAA) and organic phosphorus (lecithin) concentrations by 42.06% and 34.15%, respectively. Another PGP endophyte, YSD YN2, isolated from the same plant, exhibited improved seed germination and plant growth as a result of ARTP mutagenesis [56].

In the realm of antibacterial activity, ARTP-mediated genetic modification of *Limsolactobacillus reuteri* produced the AR-148 mutant strain [57], whose cell-free supernatant (MCFS) demonstrated potent antibacterial activity against *Escherichia coli*. MCFS disrupted cell membranes, altered biomolecules and enzyme levels, and impacted genes related to energy metabolism, oxidative stress, and cell integrity. This study sheds light on the antibacterial mechanism of MCFS against Gram-negative bacteria, presenting potential applications in the food industry.

Moreover, the ARTP-induced mutation of *Bacillus mucilaginosus* resulted in the strain BM-50, which exhibited a twofold increase in acid production capacity, leading to a 15.3% enhancement in vanadium leaching efficiency from stone coal [58]. Additionally, ARTP is employed to boost bacterial primary metabolites and generate resistant strains [59,60], further highlighting its potential as an efficient mutagenesis strategy for improving microbial performance in various industrial applications.

These findings underscore the versatility and efficacy of ARTP technology in enhancing microbial traits and performance for a wide range of applications, including agriculture, environmental remediation, and industrial processes.

**Table 1:** Achievements of ARTP mutagenesis in bacteria.

Bacteria Species	Compounds Property	Mutant Method	Time	Ability	Refs
*B. cereus*	Chitosanase	ARTP	60 s	Increase in chitosanase productivity of 3.66 times	[25]
*B. licheniformis*	Thermostable protease	ARTP	60 s	Increase in thermostable protease activity of 1.56 times	[27]
*R. sphaeroides*	CoQ10	ARTP	30 s	Increase in CoQ10 productivity of 22.1%	[28]
*R. sphaeroides*	CoQ10	ARTP	20 s	Increase in CoQ10 productivity of 25.5%	[29]
*R. sphaeroides*	CoQ10	ARTP	50 s	Increase in CoQ10 productivity of 26.9%	[30]
*R. sphaeroides*	CoQ10	ARTP	25 s	Increase in CoQ10 productivity of 18%	[31]
*R. sphaeroides*	CoQ10	ARTP	120 s	Increase in CoQ10 productivity of 16.1%	[32]
*B. amyloliquefaciens*	Alkaline phosphatase	ARTP		Increase in alkaline phosphatase activity of 4.67-fold	[33]
*E. profundum*	Protease	ARTP	120 s	Increase in protease activity of more than 20%	[34]
*B. licheniformis*	Protease and amylase	ARTP	60 s	Increase in protease and amylase activity of 143.10%	[35]
*P. algicola*	Alginate lyase	ARTP	50S	Increase in alginate lyase activity of 32.6% and 21.6%	[36]
*B. amyloliquefaciens*	α-Amylase	ARTP	30 s	Increase in α-amylase of 86.92%	[37]
*B. subtilis*	Alkaline protease	ARTP	50 s	Increase in alkaline protease activity of 23.38%	[38]
*B. subtilis*	γ-PGA	ARTP	30–180 s	Increase in γ-PGA producing of 86.8%	[39]
*C. glutamicum*	L-serine	ARTP	30 s	Increase in yield of L-serine of 66.7%	[40]
*C. glutamicum*	L-Glutamic Acid	ARTP	40 s	Increase in L-glutamic acid producing of 12.9%	[41]
*C. glutamicum*	L-glutamine	ARTP; gene editing	20 s	Increase in L-glutamine producing of 3500%	[42]
*C. glutamicum*	L-histidine	ARTP	210 s	Increase in L-histidine producing at 0.561 ± 0.016 g/L	[43]
*C. glutamicum*	L-isoleucine	ARTP	180S	Increase in L-isoleucine producing of 62.03%	[44]
*L. plantarum*	Bacteriocin	ARTP; NTG; genome shuffling	10S	Increase in bacteriocin activity of 2.35 times	[48]
*L. plantarum*	Bacteriocin	Microwave; NTG; ARTP; UV	6 s	Increase in relative bacteriostatic titers of 5.51-fold	[50]
*L. plantarum*	Bacteriocin	ARTP; MNNG; gene editing	40 s	Increase in bacteriocin yield of 103.48%	[49]
*Nonomuria* spp.	Dalbavancin precursor	ARTP; UV	30 s	Increase in dalbavancin precursor yield of 68.7%	[52]
*B. amyloliquefaciens*	Remove petroleum hydrocarbons	ARTP	30 s	Removal of petroleum hydrocarbons of 45.44%	[53]
*B. velezensis*	Remove Cr	ARTP	60 s	Increased cadmium tolerance of 400 mg/L	[22]
*P. fluorescein*	EPS	ARTP	60 s	Increase in flocculating activity of 106.48%	[54]
*Pantoea* sp.	Plant growth promoting	ARTP	50–125 s	Enhanced plant growth and antioxidative activities.	[55]
*Franconibacter* sp.	Plant growth promoting	ARTP	50–125 s	Enhanced plant growth and antioxidative activities.	[56]
*L. reuteri*	Antibacterial activity	ARTP	30 s	Showed higher antibacterial activity by 7%	[57]
*B. subtilis*	Surfactin	ARTP	24 s	Increase in surfactin yield of 334.2%	[61]
*Notoacmeibacter* sp.	HPG	ARTP	60 s	Increase in HPG yield of 94.9%	[62]
*B. mucilaginosus*	Acid	ARTP	50–70 s	Increase in acid production of about twofold	[58]
*L. acidophilus*	Acid tolerance	ARTP	60 s	75.67% and 25.78% survival rates with pH 3.0 and 2.5	[60]
*B. coagulans*	Acid/salt tolerance	ARTP	15 s	22.4% survival rate with pH 2.5 and 0.3% bile salt	[59]

## 3. Application of ARTP to Fungi

ARTP technology also plays a vital role in the application of fungi. Through ARTP-induced mutations, specific genetic characteristics can be introduced in fungi, resulting in the development of mutant strains with improved properties, ultimately leading to increased yield and quality of desired products.

### 3.1. Application to Yeast Mutation

ARTP technology has significantly contributed to the improvement and screening of yeast strains. It has proven effective in producing various yeast mutants with specific traits, such as high-yield yeast [63,64,65,66,67,68,69,70,71,72,73,74,75,76], salt-tolerant yeast [77,78], and acid-tolerant yeast [79], among others (Table 2). *Saccharomyces cerevisiae*, a well-known yeast species, holds immense importance in industries like winemaking, baking, and brewing, with a historical significance dating back to ancient times [80]. ARTP mutagenesis of this strain has resulted in numerous strains with a high production of specific compounds, including astaxanthin [63,64], lycopene [65], squalene [66], and p-coumaric acid (p-CA) [67]. Additionally, ARTP has been used to obtain fermentation strains with special robustness and high ethanol production [75], as well as strains with low acetaldehyde content [81] and acid tolerance [79].

Moreover, ARTP mutation technology has found applications in yeast genome functional research and genetic engineering. It enables the production of a large number of yeast mutants, allowing for the investigation of gene expression, metabolic pathways, and their interrelationships. For example, Cai et al. [67] identified the association of the *HTZ1* gene with tyrosine biosynthesis in *Saccharomyces cerevisiae*. Through gene knockout and ARTP mutagenesis, they obtained high-producing tyrosine mutants and conducted genome and transcriptome analysis to reveal the underlying mechanism behind the increased tyrosine production. Similarly, ARTP mutagenesis was used to generate a yellow *X. dendrorhous* mutant and the GATA transcription factor *XdWC2* was identified as crucial in controlling astaxanthin production [74]. This research laid the foundation for understanding the global regulation of astaxanthin biosynthesis and guiding the development of high-yield astaxanthin-producing strains.

The application of ARTP technology in yeast research and applications has driven significant progress, enabling the generation of diverse mutants with improved traits and shedding light on the mechanisms underlying gene expression and metabolic pathways. These advancements have broad implications for various fields of study and hold promise for further advancements in yeast-related research and applications.

### 3.2. Application to Mold Mutation

The application of ARTP technology in molds primarily focuses on inducing mutations and improving strains. By subjecting molds to ARTP, which can cause genetic mutations or variations, desirable changes in the strains can be achieved, such as increased product yield, development of functional strains and enhanced resistance (Table 2). This technology holds significant implications in various industries.

For instance, Wang et al. [82] employed ARTP and UV mutagenesis to introduce modifications in *S. peucetius* SIPI-11 and enhance the production of doxorubicin. Through screening in a doxorubicin (DXR) medium, a mutant strain (*S. peucetius* 33–24) with a remarkable DXR yield of 570 mg/L was identified, surpassing the wild-type strain’s yield of 119 mg/L. ARTP has also been effectively applied to other antibiotic-producing strains, such as natamycin [83], avilamycin [84], salinomycin [85], wuyiencin [86], and ansamitocin [87].

Additionally, ARTP mutagenesis has yielded mutant strains with improved production of diverse bioenzymes. For instance, naringinase, an enzyme with wide applications in the food, beverage, and pharmaceutical industries, saw significant productivity increases through UV-ARTP mutagenesis in Aspergillus tubingensis MN589840. High-yield mutants U1, A6, and UA13 exhibited impressive productivity enhancements [88]. Furthermore, ARTP has been successfully utilized for the production of diverse bioenzymes, including tannase [89,90], raw starch-degrading enzymes [91], thermostable xylanase [92], cellulose [93,94] and L-asparaginase [95].

ARTP mutagenesis of *Myrothecium verrucaria* has shown effectiveness in producing enzymes that efficiently remove environmental pollutants and reduce their toxicity [96,97]. Additionally, ARTP technology has demonstrated promise in agricultural yield improvement. Strains of *Aspergillus niger* v. Tiegh subjected to ARTP mutagenesis were identified as capable of efficiently degrading soil phosphorus compounds. Among them, xj120-12 is a highly efficient phosphorus-solubilizing and growth-promoting strain, significantly enhancing plant growth and yield through effective degradation of phosphorus compounds [100].

Furthermore, ARTP has opened avenues for developing biocontrol agents. Qiu et al. [98] successfully used ARTP technology to obtain fungal strains with high biocontrol potential, such as *Beauveria bassiana*. These mutant strains exhibit stronger biocontrol abilities, improved growth characteristics, and enhanced enzyme activities, making them potential biocontrol tools in complex agricultural environments.

ARTP technology is a valuable tool for enhancing filamentous fungi, inducing beneficial mutations, and improving desirable traits in mold strains. Its success is evident in increased enzyme production, enhanced lycopene synthesis, and the generation of resistant strains with improved biocontrol abilities. The application of ARTP in mold research opens new opportunities in microbial metabolic engineering and offers innovative approaches to strain improvement and utilization in various fields.

### 3.3. Application to Edible Fungi

ARTP technology is extensively applied in the breeding of edible mushrooms, which are valuable food and medicinal fungi known for their high nutritional and medicinal value. Through the use of ARTP technology, it becomes possible to modify the growth traits, yield, and quality of edible mushrooms, thereby increasing their economic worth and market competitiveness (Table 2).

For example, ARTP technology has been utilized to induce gene mutations and increase the content of flavonoids in *Phellinus baumii* [104]. Following ARTP mutagenesis and subsequent screening, a total of four mutants with high flavonoid production were obtained. Among these mutants, A67 exhibited the highest flavonoid yield, with an impressive 88.24% increase in intracellular flavonoid production. In addition to obtaining high-flavonoid-producing mutants, strains subjected to ARTP mutagenesis also yielded exceptional strains with elevated production of laccase [105], polysaccharide [106,107,108,109,110], and hispidin [111].

The fruit bodies of edible mushrooms hold significant value as both food and medicinal products. The economic worth of these fruit bodies is closely tied to their yield and quality. To enhance these characteristics, ARTP serves as an efficient mutagenesis tool that can induce mutations and then select strains with exceptional traits in mushroom resource breeding, making them more competitive in the market [112,113,114,115]. By utilizing ARTP mutagenesis, it becomes possible to enhance specific characteristics of strains, thereby endowing them with unique functionalities. For instance, Zhang et al. [116] employed 2,2-diphenyl-1-picrylhydrazyl (DPPH) scavenging capacity to screen a total of 14 mutants of *Ganoderma lucidum* with a robust antioxidant capacity from a pool of 103 mutants generated through ARTP. Through comprehensive antioxidant evaluation analysis, two mutant strains with remarkable antioxidant capacity were successfully identified.

As ARTP technology continues to advance and improve, its applications in edible mushroom breeding and improvement are expected to expand further. This technology holds great promise for enhancing the traits and characteristics of edible mushrooms, ultimately contributing to the development of more productive and high-quality mushroom varieties.

## 4. Application of ARTP in Microalgae

ARTP technology has become extensively utilized in microalgal mutagenesis research. Microalgae are highly regarded in biotechnology due to their rapid growth and efficient photosynthesis. Through ARTP mutagenesis, microalgae can be engineered to produce valuable compounds, including biofuels [117], pigments [118,119], lipids [120], and omega-3 fatty acids [121,122] (Table 3).

Mutagenesis induces alterations in metabolic pathways, enzyme activities, and regulatory mechanisms, resulting in increased product yields. One notable advantage of ARTP mutagenesis is its efficiency and rapidity in improving microalgae strains. Compared to traditional breeding methods, which rely on labor-intensive and time-consuming techniques for generating mutations, ARTP mutagenesis significantly shortens the timeframe. Fang et al. [123] utilized ARTP to induce mutations in Spirulina, with the goal of enhancing its carbohydrate yield and growth rate. From a mutant library, they screened and selected ideal mutants with increased carbohydrate content and growth rate.

Moreover, ARTP mutagenesis provides valuable insights into the synthesis mechanisms of microalgal target compounds, which underlie strain improvement. By analyzing transcriptomic and proteomic data, researchers can identify differentially expressed genes and proteins linked to increased productivity. Chen et al. [124] identified a mutant strain of *Schizochytrium limacinum* LD11 with high docosahexaenoic acid (DHA) production through ARTP mutagenesis. Molecular analysis revealed the underlying mechanisms responsible for the improved DHA yield in the mutant strain, thus enabling further breeding strategies for enhanced DHA production in *Schizochytrium*.

In conclusion, ARTP mutagenesis is a valuable tool in microalgal biotechnology, facilitating the generation of mutant libraries, accelerating strain improvement, and unraveling molecular mechanisms related to increased productivity. As ARTP technology continues to advance and integrates with other omics approaches, further breakthroughs can be anticipated in optimizing microalgae for sustainable production of valuable compounds.

**Table 3:** Achievements of ARTP mutagenesis in microalgae.

Strain	Compound/Property	Mutant Method	Time	Ability	Refs
*P. kessleri*	Biodiesel	ARTP	40 s	Increases in biomass and lipid productivity of 75% and 44%, respectively.	[117]
*S. platensis*	Astaxanthin	ARTP	70 s	Increase in astaxanthin productivity of 196%.	[118]
*H. pluvialis*	Astaxanthin	ARTP	40 s	Increase in astaxanthin yield of 61.73%.	[119]
*C. pyrenoidosa*	High yield; lipid	ARTP	40–60 s	Increases in dry weight and lipid productivity of 22.07% and 16.85%, respectively.	[120]
*Aurantiochytrium* sp.	DHA	ARTP	25 s	Increases in biomass, lipid and DHA yield of 5.77%, 16.9% and 83.2%, respectiviely.	[121]
*S. limacinum*	DHA	ARTP	20 s	Increase in DHA yield of 25.51%.	[124]
*Schizochytrium*	DHA	ARTP	60 s	Increases in DHA concentration and productivity at 41.4 g/L and 430.7 mg/L/h.	[125]
*Schizochytrium*	DHA	ARTP	40 s	Increase in DHA content of 54.1%.	[126]
*Desmodesmus* sp.	Lipid	ARTP	60–65 s	Increase in triglyceride (TAG) production of 234%.	[127]
*Desmodesmus* sp.	Lipid	ARTP	90 s	Increases in triglyceride and total lipid content of 48.98% and 114.99%, respectively.	[128]
*Desmodesmus* sp.	Lipid	ARTP	60 s	Increases in lipid production and biomass of >100% and >15%.	[129]

## 5. Future Perspectives

With continuous technological advancements and in-depth research, the application of ARTP mutagenesis in microbiology holds great promise for the future. Firstly, its high-throughput capabilities enable the rapid processing of a large number of microbial samples, leading to numerous mutations in a short time. By combining high-throughput screening and optimization approaches, the identification and selection of mutant strains can be accelerated, expediting microbial improvement and optimization. Secondly, integrating systems biology and computational modeling can further expedite the breeding of new strains. Using systems biology research approaches and computer modeling tools, the effects of ARTP mutagenesis on microbial genomes and metabolic networks can be better understood. This integration allows for the prediction and optimization of mutagenesis effects, facilitating the rapid breeding of new strains and the discovery of bioactive substances. Thirdly, combining multiple mutagenesis strategies can enhance mutation efficiency. Integrating the ARTP mutagenesis system with other microbial mutagenesis methods, such as chemical mutagens and radiation, can increase mutation diversity and effectiveness. This approach broadens the range of variations, boosts mutation frequency, and opens up greater possibilities for microbial evolution and improvement. Fourth, ARTP mutagenesis technology can be applied to environmental remediation and resource utilization. By modifying microorganisms with specialized metabolic capabilities through mutagenesis, environmental pollutants can be efficiently degraded and remediated. Additionally, improving microbial metabolic pathways can lead to the development of efficient techniques for resource utilization, such as converting waste into useful compounds or bioenergy. Lastly, ARTP mutagenesis enhances efficient production in microbial factories and industrial settings. By modifying microbial metabolic pathways through mutagenesis and regulating the expression of key genes, high-yield and efficient industrial microbial production can be achieved. This propels the development of microbial industries, fostering sustainable production and resource utilization.

In conclusion, the application prospects for ARTP mutagenesis in microbiology are extensive. Through further research and technological innovation, significant breakthroughs and applications can be anticipated in microbial improvement, bioactive substance discovery, environmental remediation, resource utilization, and industrial production. ARTP mutagenesis opens up new possibilities for research and applications in microbiology, providing novel solutions to major societal and environmental challenges.

## 6. Conclusions

ARTP technology, known for its efficiency and versatility, has gained recognition for its ability to induce gene damage and control mutagenesis under mild conditions with strict safety standards. It has found applications in bacteria, fungi, and microalgae, and holds great promise in the field of microbial improvement.

One of the primary applications of ARTP technology lies in enhancing microbial fermentation product yields. By utilizing ARTP-induced mutagenesis to introduce beneficial genetic variations, microorganisms can be optimized to synthesize specific compounds more efficiently, resulting in increased yields of fermentation products. This optimization leads to improved efficiency and economic benefits in industrial processes. Furthermore, ARTP technology enables the cultivation of microorganisms with distinct traits, which is particularly advantageous for specialized microorganisms like biodegrading bacteria. By selectively utilizing ARTP-induced mutations, specific degrading capabilities can be conferred, enhancing their potential applications across industries and environmental remediation efforts.

As ARTP technology continues to evolve and improve, its prospects across various fields are set to expand further. Its ongoing development holds the potential to bring substantial economic value and social benefits to humanity, enhancing the quality of life and contributing to public health. ARTP technology emerges as a versatile and potent tool with applications spanning multiple fields, including agriculture and environmental protection. Its continuous integration into various industries underscores its potential to address key challenges and promote human well-being on various fronts. With research and innovation continuing to unfold, ARTP technology is expected to exert a transformative influence on various scientific fields.

## Figures and Tables

**Table 2:** Achievements of ARTP mutagenesis in fungi.

Strain	Compound/Property	Mutant Method	Time	Ability	Refs.
*S. cerevisiae*	Astaxanthin	ARTP	20 s	Increase in astaxanthin yield of 4-fold.	[63]
*S. cerevisiae*	Astaxanthin	ARTP; SCRM	35 s	Increase in astaxanthin yield of 2.2- and 7.0-fold.	[64]
*X. dendrorhous*	Astaxanthin	ARTP	100–175 s	High astaxanthin production.	[74]
*S. cerevisiae*	Lycopene	ARTP	35 s	Increase in lycopene yield of 60% to 703 mg/L at shake flask.	[65]
*S. cerevisiae*	Squalene	ARTP	75 s	Increase in squalene yield of 18.4%.	[66]
*S. cerevisiae*	p-coumaric acid	ARTP	90 s	Increase in p-CA yield of 7.6-fold.	[67]
*S. cerevisiae*	Fermentation robustness	ARTP	15 s	Enhanced fermentation robustness and highest ethanol yield.	[75]
*S. cerevisiae*	Low producing of acetaldehyde	ARTP	45 s	LAL-8a produces 2.2 mg/L acetaldehyde, 88.2% less than M14.	[81]
*S. pastorianus*	NADH	ARTP; DNP	45 s	The flavor stability of beer has been enhanced.	[68]
*S. pastorianus*	RNA	ARTP	80 s	G03H8 increased RNA by 40% vs. G03.	[69]
*S. boulardii*	Selenium	ARTP	40 s	Increase in selenium yield of 56.77%.	[70]
*C. tropicalis*	Xylitol	ARTP	30 s	Increase in xylitol yield of 22%.	[71]
*Y. lipolytica*	Pyruvic acid	ARTP	420 s	Increase in PA yield of 28.9%.	[72]
*C. parapsilosis*	D-Arabitol	ARTP	140 s	The d-Arabitol yield increased by 53.98%.	[73]
*W. anomalus*	Salt tolerance	ARTP	120–150 s	Enhance resistance to a sodium chloride concentration of 18%.	[77]
*Z. rouxii*	Salt tolerance	ARTP	60 s	The RNA content increased by 160.54%.	[78]
*S. cerevisiae*	Acid tolerance	ARTP	150–210 s	The survival rate increased by 10-fold under low pH conditions.	[79]
*S. peucetiu*	Doxorubicin	UV; ARTP	—	Increase in doxorubicin production of 379%.	[82]
*S. natalensis*	Natamycin	UV; ARTP; DES	40 s	Increase in natamycin yield of 86.36%.	[83]
*S. viridochromogenes*	Avilamycin	UV; ARTP	70 s	Increase in avilamycin yield of 57.92–146.39%.	[84]
*S. fradiae*	Neomycin	ARTP	180 s	Increase in neomycin yield of 100%.	[20]
*S. albus*	Salinomycin	ARTP	360 s	Increase in salinomycin yield of twofold.	[85]
*S. albulus*	Wuyiencin	ARTP	180 s	Increase in wuyiencin production of 13.6–18.5%.	[86]
*A. pretiosum*	Ansamitocin	ARTP	—	Increase in ansamitocin production of 22.5%.	[87]
*A. tubingensis*	Naringinase	ARTP	240 s	Increase in naringinase productivity of 79.08–206%.	[88]
*A. carbonarius*	Tannase	ARTP	180 s	Enhanced the yield and properties of *A. carbonarius* tannase.	[89,90]
*P. oxalicum*	Raw starch-degrading enzyme	ARTP; EMS	500 s	Increase in RSDEs activity of 61.6%.	[91]
*M. thermophila*	Thermostable xylanase	ARTP	150–250 s	Increase in xylanase activity of 21.71%.	[92]
*T. reesei*	Cellulase	ARTP	90 s	Increase in cellulase activity of 27–46%.	[93]
*T. afroharzianum*	Cellulase	ARTP; MNNG; EMS	240 s	Increases in four different enzyme activity of 4.15- to 6.37-fold.	[94]
*A.s candidus*	L-asparaginase	ARTP	180 s	Increase in L-asparaginase activities of 2.3-folds.	[95]
*M. verrucaria*	Environmental remediation	ARTP	75 s	Increase in laccase activity of 19.04-fold.	[96]
*M. verrucaria*	Environmental remediation	UV; ARTP	85 s	Increase in oxidase producing of 106.15%.	[97]
*B. bassiana*	Biocontrol agent	ARTP	90 s	Increases in FSC and virulence of 37.4% and 32.6%.	[98]
*F. coccineum*	Fusidic acid	ARTP	120–140 s	High yield of fusidic acid in mutant strain.	[99]
*A. niger*	Phosphate-solubilizing ability	ARTP	120	The ability to efficiently degrade P compounds in soils.	[100]
*Geomyces* sp.	Red pigments	ARTP	90 s	Increase in red pigments yield of 24.4%.	[101]
*M. purpureus*	Monascus pigments	ARTP	180 s	Increase in monascus pigments production of 150%	[102]
*B. trispora*	Lycopene	ARTP	120 s	Increase in lycopene yield of 54.27%.	[103]
*P. baumii*	Flavonoids	ARTP	—	Increase in flavonoids yield of 86.67%.	[104]
*P. djamor*	Laccase	ARTP	120 s	Increase in laccase activity of 86.36%.	[105]
*H. erinaceus*	Polysaccharide	ARTP	30 s	Increase in polysaccharide yield of 23.25–47.45%.	[106,107]
*S. sanghuang*	Polysaccharide	ARTP	—	Polysaccharide yields from A130 mutants increased significantly.	[108]
*G. lucidum*	Polysaccharide	ARTP	—	Increase in mycelial polysaccharide yield of 46.14–268.57%.	[109]
*G. frondosa*	Polysaccharide	ARTP	60	Increase in mycelial polysaccharide yield of 5.90 g/L.	[110]
*P. baumii*	Hispidin	ARTP	—	Enhanced antioxidant activity.	[111]
*C. militaris*	High yield	^60^Co-γ; ARTP	150 s	Increase in fruit body yield of 32.27–36%.	[112]
*A. auriculae*	High yield	ARTP	45 s	Showed increased yield and improved quality.	[113]
*L. sordida*	High yield	ARTP	50 s	Showed improved yield and quality by 10.27% and 14.75%.	[114]
*G. frondosa*	High yield	ARTP	80–90 s	Increases in dry weight and polysaccharide content of 40.15% and 39.33%.	[115]
*G. lucidum*	High antioxidant capacity	ARTP	—	Showed significantly higher antioxidant capacity than wild type.	[116]

## Data Availability

Not applicable.
